# Analytical Investigations of Toxic *p*-Phenylenediamine (PPD) Levels in Clinical Urine Samples with Special Focus on MALDI-MS/MS

**DOI:** 10.1371/journal.pone.0022191

**Published:** 2011-08-04

**Authors:** Gero P. Hooff, Nick A. van Huizen, Roland J. W. Meesters, Eduard E. Zijlstra, Mohamed Abdelraheem, Waleed Abdelraheem, Mohamed Hamdouk, Jan Lindemans, Theo M. Luider

**Affiliations:** 1 Department of Neurology, Laboratory of Neuro-Oncology and Clinical and Cancer Proteomics, University Medical Center Rotterdam (ErasmusMC), Rotterdam, The Netherlands; 2 Department of Internal Medicine, University Medical Center Rotterdam (ErasmusMC), Rotterdam, The Netherlands; 3 Department of Pediatrics and Child Health, Soba University Hospital, University of Khartoum, Alsalam Medical Centre, Khartoum, Sudan; 4 Department of Clinical Chemistry, University Medical Center Rotterdam (ErasmusMC), Rotterdam, The Netherlands; Oxford University, United Kingdom

## Abstract

Para-phenylenediamine (PPD) is a common chromophoric ingredient in oxidative hair-dyes. In some African countries like Sudan, Egypt and Morocco but also in India this chemical is used alone or in combination with colouring extracts like Henna for dyeing of the hair or the skin. Excessive dermal exposure to PPD mainly leads to the *N*-mono- and *N,N*′-diacetylated products (MAPPD, DAPPD) by N-acetyltransferase 1 and 2 (NAT1 and 2) catalyzed reactions. Metabolites and PPD are mainly excreted via renal clearance. Despite a low risk of intoxication when used in due form, there are numerous cases of acute intoxication in those countries every year. At the ENT Hospital - Khartoum (Sudan) alone more than 300 cases are reported every year (∼10% fatal), mostly caused by either an accidental or intended (suicidal) high systemic exposure to pure PPD. Intoxication leads to a severe clinical syndrome including laryngeal edema, rhabdomyolysis and subsequent renal failure, neurotoxicity and acute toxic hepatitis. To date, there is no defined clinical treatment or antidote available and treatment is largely supportive. Herein, we show the development of a quick on-site identification assay to facilitate differential diagnosis in the clinic and, more importantly, the implementation of an advanced analytical platform for future in-depth investigations of PPD intoxication and metabolism is described. The current work shows a sensitive (∼25 µM) wet chemistry assay, a validated MALDI-MS/MS and HPLC-UV assay for the determination of PPD and its metabolites in human urine. We show the feasibility of the methods for measuring PPD over a range of 50–1000 µM. The validation criteria included linearity, lower limit of quantification (LLOQ), accuracy and precision, recovery and stability. Finally, PPD concentrations were determined in clinical urine samples of cases of acute intoxication and the applied technique was expanded to identify MAPPD and DAPPD in the identical samples.

## Introduction

**Figure 1:**
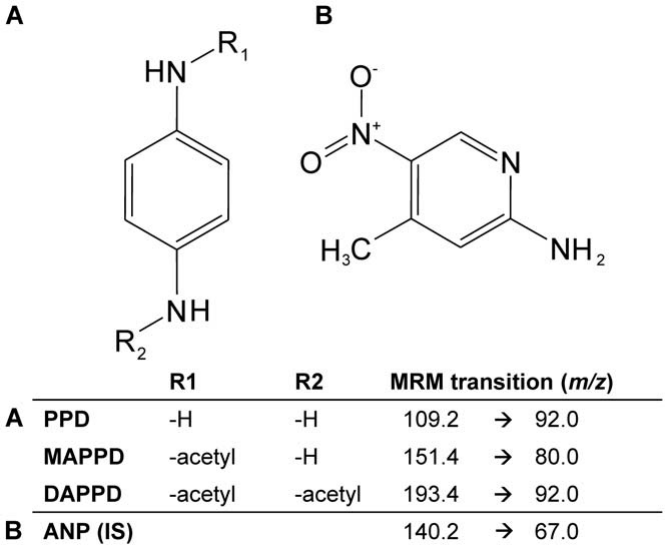
Chemical structures and respective multiple reaction monitoring (MRM) transitions of (A) PPD and its metabolites (MAPPD and DAPPD) and (B) the internal standard (IS) 2-amino-5-nitropyridine.

Since the end of the 19^th^ century the chemical *p*-phenylenediamine (PPD) has been described as a hair dye. Up till now it is used as an ingredient in more than 1000 oxidative hair-dyes in the US [1], [2]. It penetrates into the hair shaft to further undergo oxidative chemical reactions, resulting in a dark coloration of the hair. Furthermore, PPD is widely used in the dye-, photographic- or rubber industries. In addition, in many African countries like Sudan, Egypt and Morocco, PPD in its pure form or in combination with other natural colouring extracts like Henna (e.g.: Black Henna) is used for colouring of skin (e.g.: palms and soles) for cosmetic reasons [3], [4]. Unfortunately, in these developing and other newly industrialized countries (e.g.: India) there are also vast numbers of unintended and intended incidents of severe to life threatening intoxication involving this synthetic compound every year [4], [5]. Accidental poisoning is the result of a strong systemic availability by either ingestion or massive absorption of PPD via the skin. Moderate dermal exposure to PPD mainly leads to the *N-*mono- or *N,N′-*diacetylated metabolites (MAPPD and DAPPD, resp.) (Figure 1A) catalyzed by enzymatic reactions of the enzymes *N*-acetetyltransferase type 1 and type 2 (NAT1 and 2) [6]. Both enzymes are important phase II enzymes which are expressed in various organs including mainly the liver and guts (NAT2) and the skin (NAT1) facilitating the renal excretion of xenobiotics in general [6]–[8]. Other metabolites, like the mono- and diglucuronides were hypothesized but have not been confirmed yet [9]. An abnormal high exposure (intoxication) to PPD leads to saturation of the NAT1 and NAT2 enzymes resulting in the urinary excretion of the un-metabolized compound. In addition to the excretion of PPD, an increased excretion of urochromes might add to a dark color of the urine, as reported repeatedly in literature [4], [5]. Direct contact of PPD with the epidermis may lead to skin irritation or allergic reactions [10]. PPD is listed as a contact allergen by the Centers for Disease Control and Prevention (CDC) and therefore has no FDA approval for any topical formulation of cosmetics. With a LD_50_ of 80 mg/L (determined in rat) but no proven clinical evidence of causing cancer it is currently not listed as a carcinogen, although PPD and other exogenous (aryl-) amines (e.g.: aniline, which is a chemical degradation product of PPD) are being discussed as such [11], [12]. As initially mentioned, arylamines occur in the dye industry, in personal care products and cigarette smoke [13] and their metabolism has been investigated in numerous studies. Besides the aforementioned NATs, data also show a major involvement of the CYP1A family in the activation and detoxification of arylamines [12]. Furthermore, for PPD an influence on the parasympathetic nervous system has been described for animal models but has not yet been investigated in humans [14].

PPD intoxication leads within few hours after exposure to a severe clinical syndrome including laryngeal edema, rhabdomyolysis and subsequent renal failure, neurotoxicity and acute toxic hepatitis which *per se* can lead to dark coloured urine [5]. An immediate differential diagnosis is crucial to induce an appropriate and fast supportive treatment including life saving tracheostomy, antiallergic therapy and forced diuresis, as an antidote is not available yet. Late diagnosis and delayed treatment potentially lead to rhabdomyolysis and consequently to kidney failure. Early clinical manifestations of PPD intoxication symptoms potentially resemble tropical diseases like malaria, leptospirosis and hepatitis (jaundice), as well as symptoms of acute poststreptococcal glomerulonephritis or haemolytic anaemia.

The current work describes the development and validation of a Matrix Assisted Laser Desorption/Ionization tandem mass spectrometry (MALDI-MS/MS) method to quantify urinary PPD concentrations. The technique allows the possibility to measure small molecules in large sample sets in a fast, sensitive, precise and accurate manner [15], [16] and to study the correlation of severity of clinical symptoms and/or treatment progress with urinary concentrations. Moreover, this technique provides the possibility to specifically identify the metabolites MAPPD and DAPPD in urine as shown in the current study. Furthermore, a robust high performance liquid chromatography method with UV detection (HPLC-UV) was developed and tested for its feasibility. Moreover, a newly developed wet chemistry assay will allow a simple, sensitive and cost-efficient test for a fast on-site differential diagnosis of PPD intoxication in field hospitals using urine samples.

## Materials and Methods

### Ethics Statement

Sample collection at the Ear, Nose and Throat (ENT) Hospital of Khartoum was conducted by physicians or qualified personnel, educated in Good Clinical Practice (GCP) and in close collaboration with a medical doctor (EEZ) from the Erasmus MC in Rotterdam. Urine and plasma samples of cases of suspected PPD intoxication were collected for on-site diagnostic purposes following an established routine procedure. The samples used for diagnosis and subsequently for the current investigation were collected immediately after committal of the patients to the emergency unit of the clinic in Khartoum. Surplus material was anonymized prior to shipment to the Netherlands.

The investigation of remaining material was exempted from review by the Review Board of the Soba University Hospital of Khartoum, as the study followed a common clinical procedure for the investigation of rest materials. Consequently, the need for a written consent by the Ethical committee was waived, due to the fact that samples were collected on a routine basis and no study was designed prior to sample collection.

### Chemicals

All solvents were of ULC/MS grade and were bought at Biosolve (Valkenswaard, the Netherlands) unless stated otherwise. The following chemicals were bought at Sigma Aldrich (Zwijndrecht, the Netherlands): chloroform (purity of ≥99.9%), 2-amino-5-nitropyridine (ANP), *p*-phenylenediamine (PPD), α-alpha-cyano-4-hydroxycinnamic acid, formic acid (FA), diacetoxyiodo-benzene, phenol, sulfanilic acid and ammonium acetate. Sodium hydroxide and glacial acetic acid were from Merck (Darmstadt, Germany).

### MALDI-QqQ

MALDI-MS/MS analysis was conducted on a FlashQuant™ workstation combined with a 4000API triple quadrupole mass analyzer (MALDI-QqQ; Applied Bioscience/Sciex, Toronto, Canada).

All measurements were run in multiple reaction monitoring (MRM) mode unless stated otherwise. The monitored mass transitions were: PPD *m/z* 109.2→92.0; ANP *m/z* 140.2→67.0; MAPPD *m/z* 151.4→80.0 and DAPPD 193.4→92.0 (see also Figure 1A and B). The following settings were used for the MALDI-MS/MS experiments: laser frequency 1000 Hz, laser power 90%, laser raster speed 1.0 mm/s. The MALDI target plate (96*well*) was maintained at 30 V, skimmer voltage at 0 V and the CXP (collision cell energy exit potential) was at 20 V. The source gas and the CAD gas (collision gas) were kept at 10 and 12 (arbitrary units), respectively. The collision energy (CE) for PPD and the ANP as the internal standard (IS) were kept at 40 V and 25 V, respectively. Measurements were conducted with a dwell time of 10 ms. α-Cyano-4-hydroxycinnamic acid (CHCA), was used as MALDI matrix for all measurements. Data were analyzed with the FlashQuant™ 1.0 software and Analyst 1.4.2 (Applied Bioscience/Sciex, Toronto, Canada).

### HPLC-UV

Chromatographic separation was carried out on a Dionex Ultimate system using gradient elution with two solvents: solvent A, 100% water with 0.01 M ammonium hydroxide (pH 9), solvent B, 90% methanol with 10% water and 0.01 M ammonium hydroxide (pH 9). The gradient was initiated at 5% B for 4.5 min, and subsequently a linear gradient led to 95% B in 5 min, kept for 2 min and brought back to 5% B in 0.5 min. The column was equilibrated for 3.5 min. Total run time was 16 min.

A Luna phenyl-hexyl column (150×4.60 mm, 5 µm) from Phenomenex (Aschaffenburg, Germany) was kept at 20°C for the separation. PPD and ANP (IS) were monitored at a wavelength of 240 nm and 310 nm, respectively. Retention time (*Rt*) for PPD was 6.2 min and for the IS 11.0 min. Data were analyzed with the Chromeleon® v. 6.80 software from Dionex (Sunnyvale, California).

### MALDI-TOF

TOF-experiments for the exact mass confirmation of MAPPD and DAPPD and TOF/TOF experiments for their structural elucidation were performed on an Ultraflex MALDI-TOF/TOF mass spectrometer (Bruker Daltonics, Bremen, Germany) equipped with a 50-Hz nitrogen laser (337 nm). Patient samples were spotted on a 384*well* target plate with CHCA as the MALDI matrix. Mass spectra were recorded in the positive ion reflectron mode. FlexControl version 2.4 software was used to operate the mass spectrometer.

### Method validation

#### Stock solutions

A 10 mmol/L PPD stock solution was prepared by dissolving the pure compound in 10 mL of 0.1% FA in water. For the calibration standards and quality control (QC) samples, the stock solution was diluted with 0.1% FA in water.

ANP, used as the internal standard, was dissolved in water and used at a final concentration of 0.5 mmol/L.

#### Validation protocol

For the method validation pooled human urine was used from healthy controls (*n = 7*; age 19–43, mean 31.6; 4 ♂, 3 ♀). The calibration standards and QC samples were prepared by mixing 400 µL pooled drug free control urine from healthy donors with 10 µL of IS, 40 µL 2.1 M sodium hydroxide solution and 50 µL of diluted PPD stock solution.

The calibration curves consisted of a blank (pure urine), a zero sample (blank urine spiked with IS) and 7 calibration standards of PPD at the following concentrations: 50, 100, 150, 250, 500, 750 and 1000 µmol/L for the MALDI-MS/MS analysis and 150, 250, 500, 750 and 1000 µmol/L for the HPLC-UV assay. In both cases, assay linearity was calculated by using a weighting factor, previously evaluated by a comparison of the *r^2^* values and the determination of the lowest sum of absolute values of the relative errors between the calculated and the nominal value of each calibrator (data not shown). For the determination of the LLOQ for the MALDI-MS/MS, 5 separately prepared samples and 3 for the determination on the HPLC-UV system were measured at the respective concentrations. Accuracy and precision measurements were conducted with three calibration curves on one day (intraday) and on three consecutive days (interday). For the stability tests aliquots at three different concentrations (130, 400 and 800 µmol/L) were prepared in urine and kept for 15 h at room temperature in the dark, 24 h at +4°C (fridge) and 1 month at −20°C in the freezer. Recovery was determined on the MALDI-QqQ.

#### Sample preparation

Liquid/liquid extraction (LLE) was performed by adding 500 µL of chloroform to the urine sample, shaken vigorously for 20 s and centrifuged for 2 min at 16000× g to separate the organic layer from the water phase. From the chloroform layer, 400 µL were transferred to a 1.5 mL Eppendorf reaction vial. LLE was conducted twice. The combined extracts were dried in a SpeedVac at 45°C for 20 min.

The dry residue was reconstituted in a 100 µL mixture of ACN/H_2_O (50∶50 v/v). This solution was injected into the HPLC. For the MALDI-MS/MS analysis the solution was diluted (10×) with the identical ACN/H_2_O mixture. This solution was mixed 1∶1 with CHCA and 1 µL spotted on a 96*well* plate (6 spots per sample).

#### Wet chemistry assay

The wet chemistry assay was conducted based on the findings described by Verma 1979 and modified for urine sample [17]. Sample preparation (LLE) was conducted as described in the preceding paragraph with following changes to reduce drying time. Two 300 µL steps were chosen for the extraction. For each step 250 µL of the organic layer were transferred into a new vial to result in a total volume of 500 µL for the combined extracts. The extract was dried at 60°C and reconstituted with 250 µL 2 M sodium hydroxide solution. 200 µL of this solution were transferred to a 96*well* plate. The addition of 20 µL of a 1% phenol solution in glacial acetic acid and 20 µL of a 1% diacetoxyiodo-benzene in glacial acetic acid resulted in a red color of the solution with concentration dependent intensity. To prove the linear range of the calibration curve in the extract from the pooled urine (spiked at the following concentrations prior to extraction: 0, 25, 50, 100, 250 and 500 µmol/L) a UV spectrum of the highest concentration and a blank for comparison were recorded using a spectrophotometer. The absorption maximum was at 450 nm, which was used to measure the calibrants.

A change in color (in presence of PPD the solution turns red) was compared to a second extract from the identical patient urine by adding 20 µL of glacial acetic acid instead of the diacetoxyiodo-benzene glacial acetic acid solution. This reference solution was used to show the specificity of the reaction.

#### Clinical urine samples

Clinical samples (*n = 15*) were collected, documented and anonymized at the ENT Hospital of Khartoum (Sudan) in the period between December 2009 and February 2010. All patients were diagnosed with an intoxication of PPD and arrived at the clinic within 12 hours after intoxication. The time interval between urine collection and storage in the freezer at −20°C was 2–4 hours. Further urine parameters were measured and collected at the clinic. Patients were between 11 and 41 years old (mean age 25.2+/−8.8 years); 13 female and 2 male; 7 of which were in the clinic following a suicide attempt, 2 due to a homicide, 2 due to an accident and 4 due to an unknown reason.

For the preparation of the patient samples from the clinic, 400 µl of urine were mixed with 10 µl IS, 50 µl of 0.1% FA followed by 40 µL 2.1 M sodium hydroxide solution prior to LLE.

## Results and Discussion

**Figure 2:**
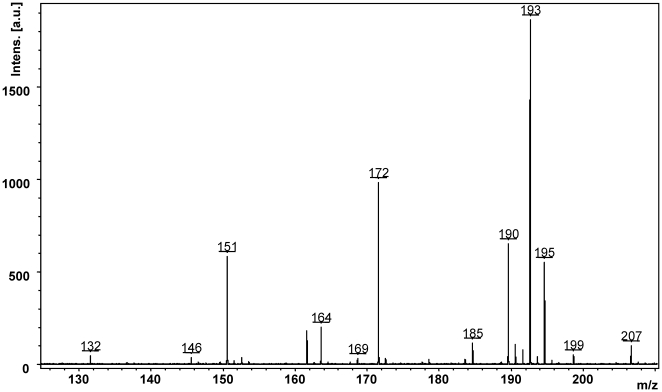
TOF spectrum (pos. mode) for the identification of two PPD metabolites MAPPD (*m/z* [M+H]^+^ 151) and DAPPD (*m/z* [M+H]^+^ 193) in patient urine. Comparative control urine did not show any signals at the corresponding *m/z* values.

The present study describes the development and validation of a MALDI-QqQ assay for the determination of PPD in human urine. Furthermore, it demonstrates a simple and fast possibility to monitor endogenous metabolites at the same measuring time in the identical samples, without the necessity of a time consuming development of a separation. In the current example calculated *m/z* values for MAPPD (*m/z* [M−H]^+^ 151) and DAPPD (*m/z* [M−H]^+^ 193) were identified by a MS1 scan on the MALDI-QqQ and in a high resolution TOF spectrum of selected patient samples (see Figure 2). Further structural information for the confirmation of the metabolites was provided by a MS2 product ion scan on the MALDI-QqQ and by a TOF/TOF spectrum with accurate masses (data not shown). Overall the loss of one or two acetyl groups (−42 Da) from MAPPD or DAPPD, the loss of ammonia (−17 Da) and presumably a hydrogen isocyanide (−27 Da) rather than hydrogen cyanide from the PPD after a loss of one amine function was observed. This conclusion is based on former studies of Burgers et al. who showed for aniline a neutral loss of 29 Da as a loss of hydrogen isocyanide [18].

It was demonstrated that MALDI-MS/MS (AB Sciex, FlashQuant™ system) provides an ideal tool to deliver ultrafast (∼8 s per sample) and valid data. These results will help to understand PPD intoxications, its metabolism and will provide the basis for further research to develop a targeted treatment scheme for the clinic. To facilitate the differential diagnosis in on-site field hospitals of developing countries we also developed a quick wet chemistry assay sensitive enough (∼25 µmol/L) to compete with the MALDI-MS/MS assay (LLOQ 50 µmol/L) and therefore suitable to detect toxicologically relevant urine concentrations by a simple color test. The development of this assay was based on a publication by Verma, which describes the detection of various arylamines in pure solutions leading to different, specific color changes of the test solution [17]. Some of these compounds potentially lead to similar color reactions, thus reducing the specificity of the developed assay. However, to the authors' knowledge there is little evidence for such compounds in human urine, which could interfere with the assay. Metabolism of these compounds is a further limiting factor for cross reactivity and concentrations of such chemicals in urine were shown to be below the detection limit described for the wet chemistry assay in the current work. Aniline, for example was shown to be below 5 µmol/L in a population based study by Kütting et al. [19].

**Figure 3:**
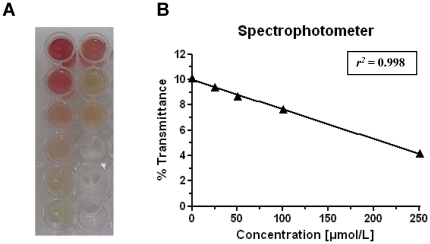
Wet chemistry assay for spiked blank and clinical urine samples. (**A**) *Left row bottom to top:* urine blank and 5 different spiked concentrations (25, 50, 100, 250 and 500 µmol/L). *Right row top three vials:* three clinical samples, which show a positive test result for PPD. (**B**) Concentration dependent transmittance (%) from 25–250 µmol/L on a spectrophotometer at 450 nm.

Figure 3A shows a blank urine sample with no color change and 5 spiked concentrations from 25–500 µmol/L and 3 patient samples with a red color of different intensity, depending on the PPD concentration. The transmittance of the standards was measured on a spectrophotometer giving a linear response (*r^2^* = 0.998) from 25–250 µmol/L (see Figure 3B). However, the highest concentration of 500 µmol/L was outside the linear range of the measurements. The LLE described in the method protocol was necessary to visualize the color change in urine samples in the presence of coloring compounds as described earlier. At this point, it should be noted that the extraction of a brown colored PPD stock solution resulted in an extract with a similar color. However, this was not observed in the dark urine samples. An explanation for the dark color of some of the urine samples potentially originates from several sources. For example, jaundice as a result of acute hepatitis can be observed in patients with PPD intoxication and leads to a steadily increasing amount of urochromes (urobilinogen, biliverdin) and bilirubin, that may add to the dark color of the urine [20]. Similarly rhabdomyolysis may occur and the excretion of myoglobin may also add to the dark color of the urine [21]. Currently it is unclear to what extent more hydrophilic, oxidized PPD derivatives form azo-groups (e.g. Bandrowski's base) and potentially also add to the color of the urine.

One of the major challenges of the current work was to find an appropriate IS simultaneously for the MALDI-MS/MS and the HPLC-UV detection. Several surrogate compounds were tested for their feasibility, including 2, 5-dihydroxybenzoic acid (DHB), aniline, acetaminophen (PCM), 4-nitro aniline and salicylamide. However, these chemicals proved to be unsuitable due to an increased background for PPD in the MRM mode, instability or showed to be unreliable. ANP was identified as a suitable IS (Figure 1B).

Another challenge was the instability of the PPD stock solutions. For initial experiments in the developmental phase of the assay PPD was dissolved in pure water. These higher concentrated stock solutions (10 mmol/L PPD) showed chemical instability (oxidation processes) in less than 24 hours, which was accompanied by a visible change of the color to red-brown. However, acidic conditions (stock solution in 0.1% FA) delayed chemical oxidation of PPD for two to three days (data not shown). Consequently, fresh stock solutions were prepared every second day including the stock solution of the IS.

### Sample preparation

During the development of the method various sample preparation steps were tested for their feasibility. These tests included protein precipitation (PPT), solid phase extraction (SPE) and liquid/liquid extraction (LLE). A simple PPT step with methanol or acetonitrile (ACN) resulted in recoveries lower than 2%, which was mainly due to a very high degree of impurities, which consequently lead to ion suppression during the MALDI-MS/MS measurements. SPE experiments (polystyrene divinyl benzene or cyanopropyl) slightly improved PPD recoveries to <10%.

For the LLE different organic solvents were used and the highest recovery was found with chloroform. The recovery was further improved when the pH of the urine sample was adjusted with NaOH to pH 9 prior to the extraction. Basic conditions push the equilibrium of protonated and unprotonated PPD into its free Brønsted-Lowry base, which consequently makes the molecule more lipophilic. A double LLE with chloroform resulted in recoveries of ∼50% (see Table 1). These results were well in line with the findings of Meyer et al. 2009 [3].

**Table 1:** Validation results of PPD in human urine.

MALDI MS/MS validation results	
**Linear range [µmol/L]**	50–1000
**LLOQ (** ***n = 5*** **) [µmol/L]**	50 (CV = 14.5%)
**Recovery (low/medium/high)**	48.7% / 48.9% / 7.7%
**Stability [% recovery]**	
**RT (15 h in dark)**	98.5%
**+4°C (24 h)**	85.6%
**−20°C (1 month)**	107.9%

MALDI-MS/MS assay: linearity, lower limit of quantification (LLOQ), recovery (at 130, 400 and 800 µmol/L) and stability after addition of formic acid.

### Method validation

#### Selectivity

Selectivity was shown for the MALDI-QqQ and the HPLC-UV. Urine samples from 7 different control donors were compared on the MALDI-QqQ and peak areas (background) for the PPD transition (*m/z* 109.2→92.0; see also Figure 1A) and the IS transition (*m/z* 140.2→67.0; see also Figure 1B) showed no significant difference. Furthermore, the identical samples were injected into the HPLC and the UV trace did not show any interfering signals at the retention time of PPD or the IS.

#### Linearity and LLOQ

The LLOQ was determined at 50 µmol/L for the MALDI-QqQ and at 150 µmol/L (see Table 1) for the HPLC-UV with a CV<15%, respectively.

**Figure 4:**
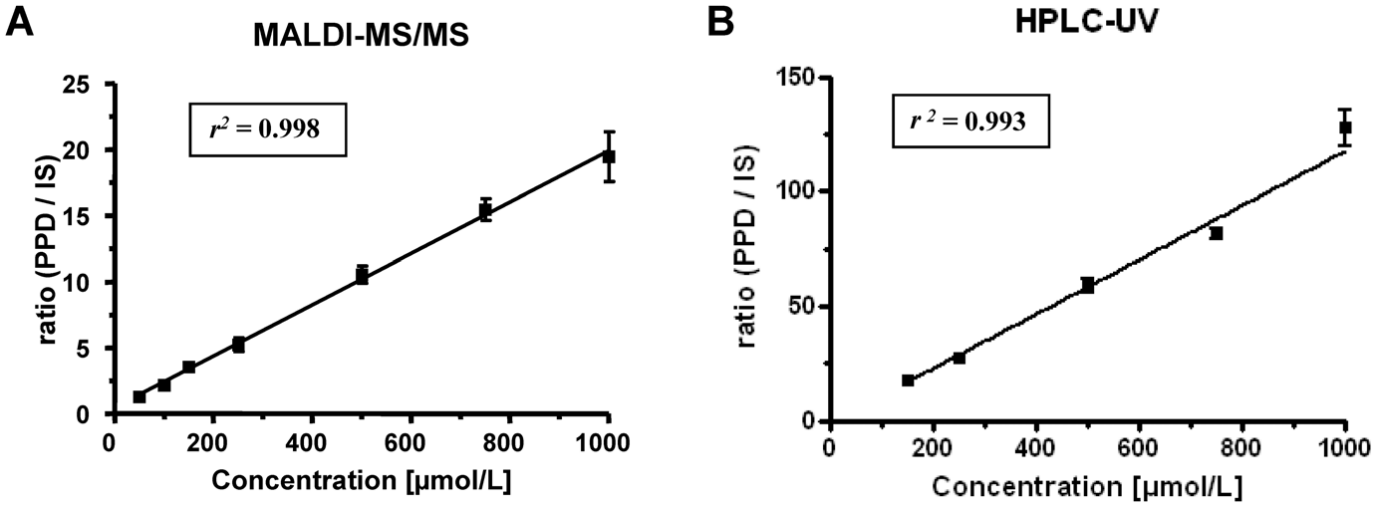
Calibration curve of PPD (A) on the MALDI-MS/MS system (50–1000 µmol/L) and (B) on the HPLC-UV system (150–1000 µmol/L). Graphs shows the mean values of (**A**) 5 and (**B**) 3 independent measurements and the corresponding *r^2^* value. Mean +/− SEM.

Good linearity on the MALDI-QqQ was repeatedly shown over the invested calibration range of 50–1000 µmol/L (see Figure 4A). The best fit for the calibration curves was achieved using a weighting factor of 1/x^2^. Higher concentrations (>1000 µmol/L) resulted in relative errors of the calculated from the nominal concentration of >25% using linear regression for the results obtained by MALDI-MS/MS.

The calibration range for the HPLC-UV was determined to be linear (see Figure 4B) from 150–1000 µmol/L. The best fit for the calibration curve was achieved using a weighting factor of 1/x^2^. Compared to the MALDI-MS/MS higher concentrations (>1000 µmol/L) were still in the linear range (data not shown).

#### Accuracy and Precision

For an assessment of the reproducibility of the assay, inter- (*n = 3*) and intraday accuracy and precision were determined on three consecutive days. The results are summarized in Table 2. With one exception for the intraday precision (24.1%) the overall results were below 20% CV at the level of the LLOQ and below 15% CV for all other values. The results for the mean accuracy were always below 17% and showed no distinct bias of the results. These data depicted a good reproducibility of the measurements on the MALDI-QqQ.

**Table 2:** Validation results of PPD in human urine.

Intra- and interday Accuracy and Precision in human urine						
	nominal conc. [µmol/L]	mean calc. conc.[µmol/L]	Accuracy (bias %)		Precision (% CV)	
			MALDI-MS/MS / HPLC-UV		MALDI-MS/MS / HPLC-UV	
**Intra-day (n = 3)**	1000	960	−3.9	2.7	24.1	10.5
	750	745	−0.6	−9.4	9.9	5.2
	500	512	2.5	2.2	8.9	8.8
	250	261	4.4	9.2	13.2	1.8
	150	157	4.5	4.7	2.4	9.4
	100	91	−8.9	n.d.	9.5	n.d.
	50	51	2.1	n.d.	16.2	n.d.

			MALDI-MS/MS		MALDI-MS/MS	
**Inter-day (n = 3)**	1000	894	−10.6		8.6	
	750	768	2.4		7.7	
	500	516	3.1		10.2	
	250	231	−7.6		13.5	
	150	175	16.3		12.5	
	100	100	0.4		8.9	
	50	48	−4.1		17.3	

Inter- and intraday accuracy and precision measurements (*n = 3* for each value). The mean relative error of each concentration is expressed as % bias and the reproducibility is depicted as the coefficient of variation (% CV). Intraday accuracy and precision were also determined on the HPLC-UV system.

The repeated measurement (*n = 3*; intraday) of the calibration curve using HPLC-UV resulted in absolute errors of the accuracy (bias) below 10% and a CV of below 11% for the precision (Table 2).

#### Recovery

Recovery was determined at three different concentrations of the calibration range to determine if the concentration of the PPD in the sample had an influence on the distribution between the hydrophilic urine and the lipophilic chloroform layer during the extraction. Recoveries were found as mentioned earlier to be about 50%, while a slight but not significant increase was observed at higher concentrations (800 µmol/L) (see Table 1).

#### Stability

As mentioned earlier pure PPD solutions were found to have an increased stability in acidic conditions. The pH of human urine typically ranges from 4.5 to 7.5. The stability of spiked urine samples was tested at physiological pH at room temperature, +4°C and at −20°C. Results are summarized in Table 1. The most striking result was that PPD recoveries dropped when the urine samples were kept at +4°C for 24 hours. There was roughly a loss of 15%, strongly emphasizing that during sample collection in the clinic urine samples need to be frozen at −20°C immediately after collection.

#### Analysis of clinical samples

To the authors' knowledge, this is the first report of the measurement of PPD and its metabolites in human urine samples from clinical cases of intoxication. For reasons of speed, sensitivity, selectivity and the possibility to simultaneously scan for the PPD metabolites, patient measurements were conducted using MALDI-MS/MS. For the HPLC-UV application a good linearity with a LLOQ of 150 µmol/L was shown (Figure 4B), allowing the accurate detection of two of the 15 investigated urine samples.

**Figure 5:**
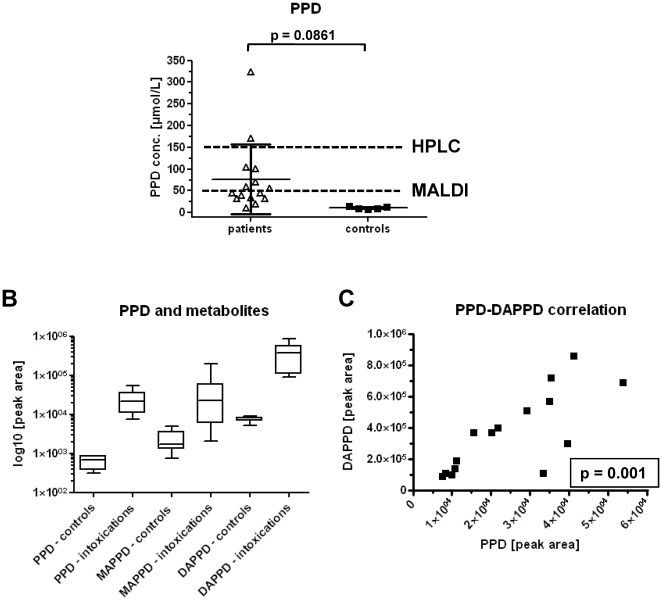
MALDI-MS/MS measurements of urine samples from patients with suspected PPD intoxication and controls. (**A**) Measured PPD concentrations in clinical urine samples (*n = 15*); dotted lines show the LLOQ of the MALDI-MS/MS and the HPLC-UV application, resp. (**B**) Box-and-whisker blot with the 5–95 percentiles for the comparison of PPD, MAPPD and DAPPD peak areas in drug free control urine (*n = 5*) and clinical samples of intoxication (*n = 15*). (**C**) Data correlation (*r^2^* = 0.7618) of PPD and DAPPD in patient urine (*n = 15*). Respective p-values are given in the graphs.

Using the MALDI-MS/MS approach urine samples showed concentrations up to 324 µmol/L and a mean value of 76 µmol/L (Figure 5A). Investigations of the metabolites on the same instrument showed significantly increased values for PPD, MAPPD and DAPPD compared to the control urine samples. Results are displayed in a box-and-whisker plot with the 5–95 percentiles (Figure 5B). Values for the MAPPD showed a larger variance, including 4 patients with no measurable MAPPD, but DAPPD values. Consequently, DAPPD could be nicely correlated to PPD (r^2^ = 0.7618, p = 0.001) (see also Figure 5C). Although for instance Nohynek and colleagues investigated the influence of the NAT2 genotype (slow and intermediate acetylators) after dermal exposure and saw no significant difference in the metabolite profile of 8 human subjects due to the combined metabolism of the NAT1 and 2 [22], this matter has not yet been investigated after PPD ingestion, where the primary metabolism will be more influenced by the NAT2 due to its location in the guts and liver [23].

### Conclusion

The current work describes the systematic approach for an in-depth investigation of PPD intoxication, treatment effects and metabolism. As a first important step, the current work shows a simple and quick assay which will greatly support physicians in hospitals to diagnose patients with PPD intoxication. In addition to previously described unspecific symptoms, this assay allows for a fast and cost-efficient identification of the toxin itself in urine samples by a simple color change. Handling needs no further technical knowledge and results can be obtained on-site within 30 minutes. With a detection limit of about 25 µmol/L this assay is roughly as sensitive as the MALDI-QqQ assay, but lacks the specificity of this advanced technique and has a shorter dynamic range. A reliable diagnosis is the first step for a targeted treatment, which we aim to establish with the help of future investigations of the intoxication. University hospitals like the one in Khartoum are the ideal surrounding to collect urine and plasma samples from cases of identified PPD intoxication. The developed MALDI-MS/MS application combines a simple sample preparation with a sophisticated measuring technique, which proved to be an ideal tool to accurately monitor PPD and both metabolites simultaneously. Compared to a common HPLC-UV method (analysis time 16 min), this mass spectrometry assay is much faster (analysis time ∼8 s) and 3 times more sensitive (see comparison in Figure 5A), which will be needed in the future to evaluate pharmacokinetic studies regarding PPD. Furthermore, this technique is more specific, robust and does not depend on any kind of separation. Moreover, preliminary data show that the method can easily be expanded on plasma measurements as we could identify mainly DAPPD in respective plasma samples (data not shown). We were not able to identify PPD in plasma which is well in accordance to the literature where PPD plasma clearance is described to appear in less than 9 hours, measured in rabbits after a single dose of 15 mg/kg body weight [24].

The investigated samples are the first report of PPD levels from patients with a diagnosed PPD intoxication. The data clearly show the excretion of un-metabolized PPD which correlates with DAPPD levels and therefore give the rational for further investigations in urine samples. The simultaneous determination of the toxin and the metabolites can be used to determine pharmacokinetics in combination with the development of a first-line treatment before kidney failure occurs [4]. Despite a few publications regarding measurements of PPD and its metabolites in biological samples [1], [3], [24], [25] there is a clear lack of precise information on PPD levels during the progression of an intoxication, with and without treatment. Moreover, there are no defined critical or target values during diagnosis and therapy. Furthermore, these studies will greatly facilitate the possibilities for the identification of an appropriate antidote.
